# The contribution of electrical synapses to field potential oscillations in the hippocampal formation

**DOI:** 10.3389/fncir.2014.00032

**Published:** 2014-04-03

**Authors:** Anna Posłuszny

**Affiliations:** Laboratory of Neuroplasticity, Department of Molecular and Cellular Neurobiology, Nencki Institute of Experimental Biology, Polish Academy of SciencesWarsaw, Poland

**Keywords:** electrical synapse, gap junctions, field potential oscillations, neuronal synchronization, interneurons, fast spiking cells, parvalbumin interneurons

## Abstract

Electrical synapses are a type of cellular membrane junction referred to as gap junctions (GJs). They provide a direct way to exchange ions between coupled cells and have been proposed as a structural basis for fast transmission of electrical potentials between neurons in the brain. For this reason GJs have been regarded as an important component within the neuronal networks that underlie synchronous neuronal activity and field potential oscillations. Initially, GJs appeared to play a particularly key role in the generation of high frequency oscillatory patterns in field potentials. In order to assess the scale of neuronal GJs contribution to field potential oscillations in the hippocampal formation, *in vivo* and *in vitro* studies are reviewed here. These investigations have shown that blocking the main neuronal GJs, those containing connexin 36 (Cx36-GJs), or knocking out the Cx36 gene affect field potential oscillatory patterns related to awake active behavior (gamma and theta rhythm) but have no effect on high frequency oscillations occurring during silent wake and sleep. Precisely how Cx36-GJs influence population activity of neurons is more complex than previously thought. Analysis of studies on the properties of transmission through GJ channels as well as Cx36-GJs functioning in pairs of coupled neurons provides some explanations of the specific influence of Cx36-GJs on field potential oscillations. It is proposed here that GJ transmission is strongly modulated by the level of neuronal network activity and changing behavioral states. Therefore, contribution of GJs to field potential oscillatory patterns depends on the behavioral state. I propose here a model, based on large body of experimental data gathered in this field by several authors, in which Cx36-GJ transmission especially contributes to oscillations related to active behavior, where it plays a role in filtering and enhancing coherent signals in the network under high-noise conditions. In contrast, oscillations related to silent wake or sleep, especially high frequency oscillations, do not require transmission by neuronal GJs. The reliability of neuronal discharges during those oscillations could be assured by conditions of higher signal-to-noise ratio and some synaptic changes taking place during active behavior.

## INTRODUCTION

Electrical synapses, from the morphological point of view, belong to a group of membrane junctions that exist in almost all animal tissues and are referred to as gap junctions (GJs). A GJ is an area of close apposition of adjacent cell membranes where an assembly of channels that pierce both cellular membranes is located, providing direct contact between the interiors of the cells. The direct transmission of electrical potentials between cells was first discovered in cardiac ganglion cells and motoneurons in crustacean and fish (Furshpan and Potter, 1957, 1959; Watanabe, 1958; Bennett et al., 1959). GJ’s were later identified as the possible structures at the cellular membrane that establish a path for electrical transmission (Bennett et al., 1963; Robertson, 1963). Data showed that electrical coupling in fish motoneurons was a source of simultaneous activity of a large portion of the effector cells: abdomen flexor muscles providing movements of the tail in crayfish (Furshpan and Potter, 1957) or mucous glands of the skin (Bennett et al., 1959).

In the 1970s, neuron coupling GJ’s were confirmed in the mammalian brain (Sloper, 1972; Sotelo and Llinás, 1972). This discovery raised many fundamental questions, especially regarding their possible function. At that time the most obvious idea about the function of GJ’s in the brain was their involvement in neuronal synchronization. It seemed that coherence of synchronous activity in a portion of neurons could be achieved by the direct spreading of excitatory potentials between them. Therefore, GJs became a new player in the mechanisms underlying the generation of field potential oscillations. Since GJ’s establish direct intercellular connection between neurons, they may provide a rapid way for transmission of electrical potentials. In that regard they have been considered especially well suited to participate in the mechanism of oscillations in high frequency bands, such as the high frequency oscillations also referred to as ripples (100–200 Hz) and gamma rhythm (40–100 Hz). Both of these oscillatory patterns in subsequent years have been recognized as a reference for information coding (Buzsáki, 1989; Lisman and Idiart, 1995; Lee and Wilson, 2002). Another hippocampal field potential oscillatory pattern related to information processing, and the last to be investigated from the point of view of GJ function was the theta rhythm (3–10 Hz; Buzsáki, 1989; O’Keefe and Recce, 1993; Skaggs et al., 1996).

The most pioneering hypothesis on the contribution of GJs to the mechanism of high frequency oscillation generation in the CA1 area of the hippocampus was proposed by Draguhn et al. (1998) and developed by Traub and Bibbig (2000). In their model, Traub and Bibbig (2000) assumed that GJs involved in high frequency activity should be localized in the neuronal membrane compartment where active conductance exists. However, direct potential exchange through GJs in areas of active membrane conductance between high frequency discharging neurons could result in asynchronous activity. The model therefore postulates that high frequency oscillations are generated by electrically coupled axons of pyramidal cells, as they generate action potentials with low frequency. According to the model, each axon should on average connect to more than one other axon and action potential generated in one axon could trigger a discharge in axons of coupled cells. Another possibility is that interneurons, which are in minority among the neurons of the hippocampal formation (only about 10% of neuron number) but have been shown to shape the activity of the projecting cells (Buzsáki and Chrobak, 1995; Cobb et al., 1995; Ylinen et al., 1995; Whittington and Traub, 2003; Le Van Quyen et al., 2008), could synchronize their own activity by GJs and provide synchronization of numerous projecting cells. Alternatively, direct transmission by GJs could have a minor influence on field potential oscillations in the hippocampal formation, as fast transmission in the network of interneurons connected by chemical synapses has been proposed to be very effective in synchronizing neuronal activity (Wang and Buzsáki, 1996; Szabadics et al., 2001; Bartos et al., 2002, 2007; Hu et al., 2011). In order to determine the significance of GJs to particular oscillatory patterns of the hippocampal field potential, studies using GJ blockers or genetically modified knock out mice for the Cx36 gene (a gene that codes protein subunits specific for GJ coupling neurons) have been undertaken. Concurrently, investigations on the properties of transmission through GJ channels as well as GJ functioning in pairs of coupled neurons have been conducted. The results of experiments with field potential recordings under condition of Cx36-GJ blockade indicate that neuronal GJs contribute to active behavior-related theta and gamma rhythms, but not to high frequency oscillations. It is proposed here that data on properties of GJ transmission between pairs of neurons indicate a possible explanation of specific GJs involvement into distinct field oscillatory patterns.

## GAP JUNCTIONS IN THE MAMMALIAN BRAIN

Gap junction are not homogeneous in their electrical conductance. The specific features of particular types of GJ depend on the protein subunits, or connexins (Cxs), from which the channels are formed (Bevans et al., 1998; Ek-Vitorín and Burt, 2005). Expression of connexins differs among distinct tissues and cellular populations (Harris and Locke, 2009). In the adult brain, expression of connexins Cx26, Cx30, Cx32, Cx36, Cx43, and Cx45 have been documented (see **Table 1**). Communication between neurons appears mainly associated with connexin Cx36, which is called the main neuronal connexin (Rash et al., 2000, 2001a,b). The Cx36 subunit composes homotypic channels only, which means that channels contain the same subunit type (Al-Ubaidi et al., 2000). Other connexins of the brain, such as Cx26, Cx30, Cx32, Cx43, and Cx45, couple glial cells. Astrocytes are coupled by GJs built from connexins Cx26, Cx30, and Cx43 (Nagy et al., 2001; Rash et al., 2001a; Condorelli et al., 2002). Astrocytes also establish GJ connection with oligodendrocytes. These heterogeneous GJ channels are formed by Cx26, Cx30, or Cx43 subunits at the astrocyte membrane and by Cx32 or Cx45 at the oligodendrocyte site (Kunzelmann et al., 1997; Rash et al., 2001a). Sparse coupling between oligodendrocytes may be supported by connexins Cx32 or Cx45 (Kunzelmann et al., 1997).

**Table 1:** Connexin types in the hippocampal formation.

Connexin type	Coupled cell types	Source
Cx26	Astrocyte–astrocyte Astrocyte–oligodendrocyte (at the astocyte membrane)		
Cx30	Astrocyte–astrocyte Astrocyte–oligodendrocyte (at the astocyte membrane)	Condorelli et al. (2002) Nagy et al. (2001) Rash et al. (2001a)
Cx43	Astrocyte–astrocyte Astrocyte–oligodendrocyte (at the astocyte membrane)	Theis et al. (2003) Wallraff et al. (2004)	
Cx32	Astrocyte–oligodendrocyte (at the oligodendrocyte membrane)	Rash et al. (2001a)
	Astrocyte–oligodendrocyte (at the oligodendrocyte membrane) Oligodendrocyte–oligodendrocyte	Kunzelmann et al. (1997)
Cx45	Astrocyte–oligodendrocyte (at the oligodendrocyte membrane) Oligodendrocyte–oligodendrocyte	Kunzelmann et al. (1997)
Cx36	Interneurons (connections within particular class): basket cells, axoaxonic bistratified cells	Baude et al. (2007)
	Interneurons (specifically)	Kosaka and Hama (1985)

It is well known that GJs couple interneurons in the brain and that these interneurons mainly belong to the same population (Sloper and Powell, 1978; Galarreta and Hestrin, 1999; Gibson et al., 1999; Deans et al., 2001; Rash et al., 2001a,b; Szabadics et al., 2001; Fukuda and Kosaka, 2003; Hestrin and Galarreta, 2005; Baude et al., 2007; Ma et al., 2011). Only a few examples of connections between heterogeneous neurons have been found (Gibson et al., 1999, 2005; Venance et al., 2000). As an exception, neurogliaform cells establish GJs with various types of interneurons (Simon et al., 2005).

The conductance of the Cx36 connexin channel reaches a level of 10–15 pS (Srinivas et al., 1999). To give a point of reference, the above values of unitary conductance are the lowest among all mammalian connexin channels. Most connexin channels have high unitary conductance of up to 300 pS (Harris, 2001). The low conductance of neuronal connexin channels is an example of a particular connexin adjustment to the functional character of the cell type they are localized in (Cruikshank et al., 2005). In neuronal tissue, the Cx36 channel unitary conductance is comparable to that of some low conductance α-amino-3-hydroxy-5-methyl-4-isoxazolepropionic acid (AMPA)-receptor channels that amounts to ~10 pS (Dingledine et al., 1999). Fukuda et al. (2006) calculated that in normal conditions only 2–5% of channels in the GJ plaque are open. A number of factors, such as intracellular pH, phosphorylation, calcium ions concentration, and metabolic pathway messengers have been shown to modulate GJ transmission (Spray et al., 2002; Lampe and Lau, 2004; Moreno, 2005; González-Nieto et al., 2008; Zsiros and Maccaferri, 2008; Pereda et al., 2013). Srinivas et al. (1999) assumed that the low level of unitary conductance of Cx36 provides precise control of the level of transmission through GJs. Control of GJ transmission can be performed by modulating the opening and closure of GJ channels in the response to changes in intracellular environment or to signal molecules. However, it can also be regulated by way of internalization of some number of channels from the cellular membrane or decreasing the number of channels that are incorporated into the membrane. The process could be relatively fast as connexin channels have very short half-life in the cellular membrane (~1.5 h).

## TOOLS FOR EXPERIMENTAL MODULATION OF GJ TRANSMISSION

In the majority of experiments GJ transmission was inhibited by pharmacological agents, such as carbenoxolone and octanol. Another compound that was used to block GJs was the anesthetic agent halothane. These agents have a wide spectrum of actions and can affect different types of GJs independently of subunit composition (Spray et al., 2002). This means that in the brain they affect not only GJs coupling neuronal cells but also those coupling glial cells. Moreover, the most frequently use carbenoxolone, exerts some non-specific effects. It was shown to alter intrinsic membrane properties (Rouach et al., 2003), block long-term potentiation (LTP; Chepkova et al., 2008), reduce excitatory postsynaptic currents (EPSCs) mediated by AMPA receptors, reduce inhibitory postsynaptic currents (IPSCs) mediated by GABA_A_ receptors (Tovar et al., 2009). Carbenoxolone inhibits activity of the enzyme 11 beta-hydroxysteroid dehydrogenase, which catalyzes the conversion of corticosterone or cortisol to their inert form (Rajan et al., 1996). As glucocorticoid receptors exist in the hippocampus and they have been shown to exert an effect on the theta rhythm (Rajan et al., 1996; Murphy et al., 1998) that could be the other possible way carbenoxolone may influence field potential oscillations.

From the point of view of GJ contribution to the neuronal synchrony, the most valuable research efforts are those where coupling between neurons was specifically blocked. Quinine has been used in a few studies, and was shown to block specifically those channels built from connexins Cx36 and Cx50 (Gajda et al., 2005; Nassiri-Asl et al., 2008). As connexin Cx50 is not expressed in the brain, local injection of quinine into brain structure blocks Cx36-GJs (Srinivas et al., 2001). A quinine derivative, mefloquine, also specific for Cx36- and Cx50-GJs, was shown to be more potent than quinine (Cruikshank et al., 2004; Behrens et al., 2011). However, application of quinine and mefloquine is also not free from non-specific effects. Quinine is known as an inhibitor of potassium channels (Henquin, 1982; Smirnov et al., 1999; Päsler et al., 2007). After mefloquine administration, Behrens et al. (2011) observed reduced pyramidal cell firing and prolongation of the afterhyperpolarization following an action potential.

Due to the non-specific action of GJ blockers, experiments with their use need to be carefully controlled and interpreted with caution. One of the possible solutions is inhibition of the non-specific targets of GJ blocker action. For example, several studies applied carbenoxolone concurrently with antagonists of chemical transmission (Schweitzer et al., 2000; Yang and Michelson, 2001; Gigout et al., 2006; Zsiros et al., 2007; Chapman et al., 2009; Kraglund et al., 2010).

Another approach to block GJ transmission specifically in neurons and eliminate non-specific effects is the use of Cx36 gene knockouts (Cx36KO; Hormuzdi et al., 2001; Buhl et al., 2003; Pais et al., 2003). However, there is a risk that compensatory processes may occur in mutants. While there are known examples of compensation when the lack of certain connexins is genetically inherited (Hombach et al., 2004), other connexins seem to be indispensable, even in tissues where many connexins are expressed in the same cell type (Richard et al., 2002). In order to assess the possible compensation in Cx36KO mice, measurements of mRNA for other related proteins (connexins: Cx30.2, Cx37, Cx43, Cx45, pannexins: PANX1, PANX2, and GABAA receptor α1 subunit) by quantitative real-time PCR were performed, and no difference between Cx36KO vs. control group was observed. However, compensation may also be accomplished by functional plasticity (De Zeeuw et al., 2003; Voss et al., 2010).

## GAP JUNCTION TRANSMISSION BETWEEN INTERNEURONS

The rate of transmission through GJs remains under great influence from the conductance properties of the cell membrane in which they are localized (Zsiros et al., 2007; Pereda et al., 2013). The majority of gap junctions exist within the areas of soma and proximal dendrites within a distance of 50 μm from the soma (Szabadics et al., 2001; Fukuda and Kosaka, 2003). However, they have been observed as far as 380 μm from the soma (Fukuda et al., 2006). Within the parts of the cell membrane with passive conductance, signals transmitted through GJs are delayed and attenuated. Efficiency of GJ transmission is measured as the ratio of potential resulting from GJ transmission in the postsynaptic cell to the potential generated in the presynaptic cell. This ratio is referred to as coupling coefficient. A coupling coefficient at the level of 0.1 was recorded in young brain neocortical neurons (Gibson et al., 2005). However, the coupling coefficient of GJ-coupled interneurons in the adult brain neocortex or juvenile hippocampus ranges from 0.035 to 0.05 for slow dynamic signals, e.g., subthreshold potentials or slow phases of the action potentials (Szabadics et al., 2001; Galarreta and Hestrin, 2002; Zsiros and Maccaferri, 2005; Zsiros et al., 2007). Fast potential changes initiated in one cell are greatly attenuated when passing through gap junction channels: the coupling coefficient for a spike is approximately 0.005 (Galarreta and Hestrin, 2002; Zsiros and Maccaferri, 2005). Signals of slow dynamics have a higher coupling coefficient because more ions can flow from cell-to-cell within a longer time under conditions of passive conductance and small throughput of electrical synapse. As a consequence of their transmission properties and localization, electrical synapses in the brain promote signal transmission of low frequency and are described as a low pass filter. Draguhn et al. (1998) proposed that low pass filtering of GJs could be overcome if they would be localized in domains of the neuronal membrane where active conductance exists, i.e., the axon. Particularly, putative axo-axonal GJs were considered to be localized in excitatory projecting cells (Draguhn et al., 1998; Traub and Bibbig, 2000). Attempts to verify the existence of electrical synapses between pyramidal cells were taken up by Mercer et al. (2006) and Hamzei-Sichani et al. (2007). However, morphological evidence was only provided for sparse close appositions between pairs of mossy fibers within the CA3 hippocampal area (Hamzei-Sichani et al., 2007).

Data indicate that in the hippocampal formation, the interneurons mainly involved in generation of gamma rhythm and ripples are three types of parvalbumin expressing (PV^+^) interneurons: PV^+^ basket cells (as opposed to basket cells not expressing parvalbumin), bistratified cells, and axo-axonic cells. Specifically, PV^+^ basket cells and bistratified cells generate discharges phase-locked to the oscillation cycle of gamma rhythm and ripples in each cycle of the oscillations (Hájos et al., 2004; Klausberger et al., 2004; Gloveli et al., 2005; Tukker et al., 2007; Bartos and Elgueta, 2012). Even more interneuron classes show activity phase-locked to the field potential theta rhythm. Among them are: PV^+^ basket cells, bistratified cells, axo-axonic cells, and oriens-lacunosum moleculare (O-LM) cells (Klausberger et al., 2003, 2004; Gloveli et al., 2005). O-LM cells seem to be specifically related to theta rhythm generation, as they have a strong intrinsic single-cell theta rhythm (Maccaferri and McBain, 1996). However, data indicate that connections between O-LM cells are not sufficient to synchronize their network activity (Rotstein et al., 2005). It seems that, while O-LM cells produce theta rhythmicity, reciprocal connections between O-LM cells and fast-spiking (FS) cells are required to synchronize signals produced by O-LM cells into field potential theta rhythm (Rotstein et al., 2005).

**FIGURE 1:**
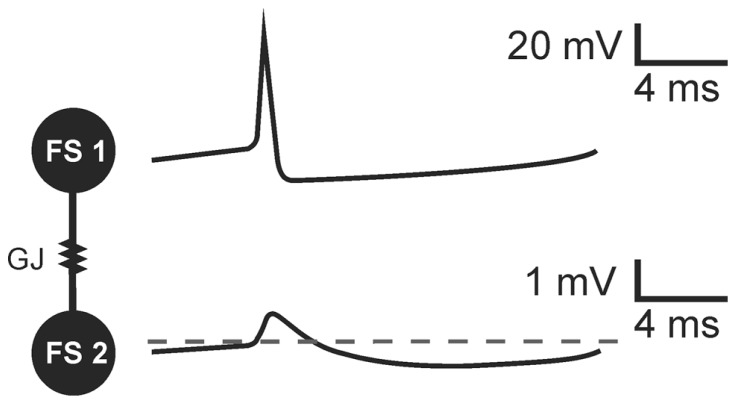
**The transmission of threshold excitation through a GJ containing Cx36 subunit in a pair of FS interneurons.** Image based on original data from Gibson et al. (2005). In response to action potential generated in a presynaptic cell (FS 1), a biphasic potential is mediated through the GJ to a postsynaptic FS cell (FS 2). Due to low-pass filtering of Cx36-GJs, fast spike is more attenuated than slow afterhyperpolarization. The biphasic potential in a postsynaptic cell is composed of a small-amplitude short depolarizing phase and long-lasting hyperpolarization in a postsynaptic FS cell.

PV^+^ basket cells, bistratified cells, and axo-axonic cells present electrophysiological characteristics of FS cells. They have been shown to establish connections by gap junctions within their groups. PV^+^FS cells produce two-phase action potentials which are composed of a very fast depolarizing phase (spike) and subsequent long-lasting afterhyperpolarization (Galarreta and Hestrin, 2002; Pawelzik et al., 2003; Gibson et al., 2005; Papp et al., 2013). In a pair of FS cells coupled by GJs, two-phase action potential generated in one neuron results in a biphasic potential in the coupled cell (Galarreta and Hestrin, 2001; Gibson et al., 2005; **Figure 1**). However, due to Cx36-GJ low pass filtering, fast signals are strongly attenuated and a spike in the presynaptic cell results in a small amplitude, short depolarization in a postsynaptic cell. Subsequent slow afterhyperpolarization is less attenuated. Therefore, in the effect of the presynaptic FS cell discharge, coupled neurons receive a potential composed mainly of the hyperpolarization beginning with a small depolarizing deflection (Galarreta and Hestrin, 2001; Gibson et al., 2005; **Figure 1**).

### THE MECHANISM OF SYNCHRONY DETECTION

**FIGURE 2:**
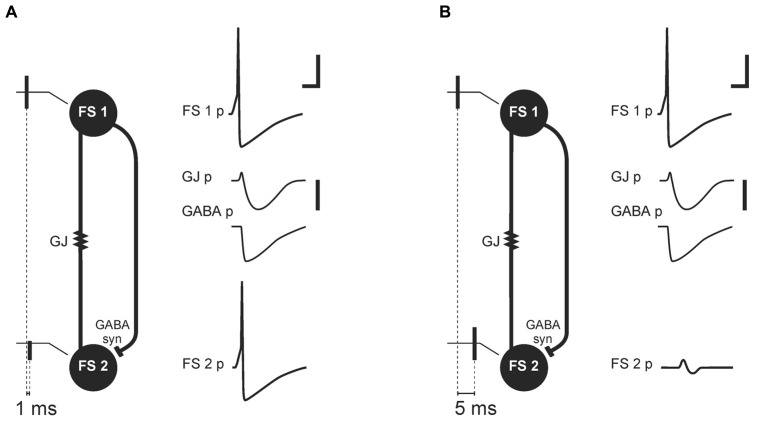
**Mechanism of synchrony detection, as described by Galarreta and Hestrin (2001)**. Image based on their original data (Galarreta and Hestrin, 2001). Schemas **(A)** and **(B)** represent two FS cells (FS 1 and FS 2) connected by electrical (gap junction containing Cx36 subunit, GJ) and a GABAergic synapse (GABA syn). FS 1 and FS 2 receive afferent threshold input with a delay of 1 ms **(A)** or 5 ms **(B)**. In response to the threshold input, FS 1 generates an action potential (FS 1 potential, FS 1 p). The GJ and GABAergic synapse mediate potentials (GJ p, GABA p) from FS 1 to FS 2. **(A)** Afferent threshold inputs to FS 1 and FS 2 succeed one another by 1 ms. FS 2 generates an action potential (FS 2 potential, FS 2 p) before inhibition mediated by the GJ and GABAergic synapse. **(B)** Afferent threshold inputs to FS 1 and FS 2 succeed one another by 5 ms. Excitation in FS 2 is attenuated by hyperpolarization mediated through the GJ and GABAergic synapse (FS 2 p). Scale bars: 20 mV for FS cells membrane potential, 1 mV for potentials mediated by electrical and chemical synapse, 5 ms.

Galarreta and Hestrin (2001) proposed that transmission through GJs in PV^+^FS cell networks could be a part of a synchrony detection mechanism (**Figure 2**). This mechanism relies on two kinds of connections between PV^+^FS cells: GJs and axonal collaterals ending with GABAergic synapses (Cobb et al., 1997; Galarreta and Hestrin, 2001; Bartos et al., 2002; Chamberland and Topolnik, 2012). When one FS neuron generates action potential in a pair of FS cells interconnected by GJs and GABAergic synapses, the coupled neuron responds with an initial small and short depolarization, mediated by GJs, and subsequent hyperpolarization, mediated by both GJs and GABAergic synapses. Such an interconnected FS cell network is preferential for synchronous excitatory inputs to FS cells. While synchronous excitatory inputs, or inputs received within a 1-ms time-window, are enhanced by the initial depolarizing phase of potential mediated by GJ, delayed inputs are attenuated by inhibition which is mediated by both GJs and GABAergic synapses (Galarreta and Hestrin, 2001). Therefore, discharge probability in FS cells excited after delay decreases. These data indicate that a primary source of neuronal synchronization is simultaneous excitation from afferent inputs, while attenuation of non-coherent signals provided by GJs and GABAergic connections between FS interneurons is a secondary contribution.

### DIFFERENCE IN ELECTRICAL VS. GABAergic SYNAPSE CONTRIBUTION TO NEURONAL SYNCHRONY

**FIGURE 3:**
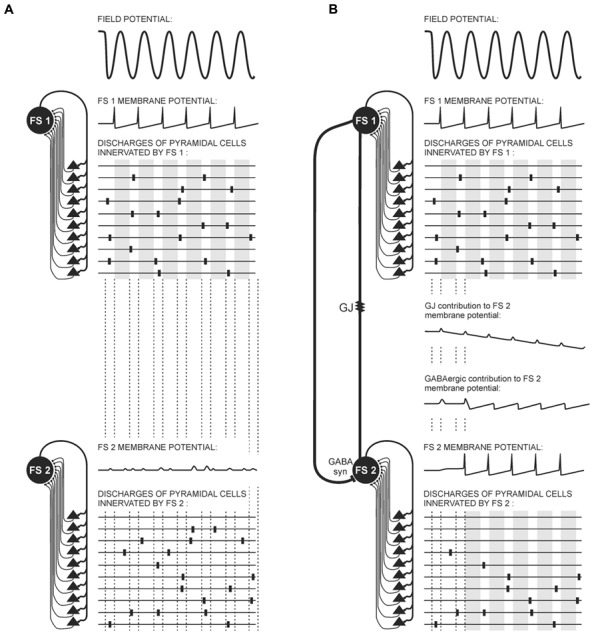
**Possible contribution of electrical and GABAergic synapses to FS interneuron and pyramidal cell activity during gamma oscillations.** Diagrams represent connections between FS cells (FS 1, FS 2) and the pyramidal cells (black triangles), and three aspects of the electrical activity of these cells: local field potential (top), FS cell membrane potentials, and schemas of pyramidal cell discharges. The top part of the diagram is identical in **(A)** and **(B)**: a portion of pyramidal cells provide coherent threshold input to FS 1. In response to this input, FS 1 generates rhythmical action potentials imposing a time-frame on pyramidal cell activity. Within this time-frame, the time-windows when pyramidal cell activity is not attenuated (white stripes) alters with the time-windows when pyramidal cell activity is attenuated (gray stripes). **(A)** FS 1 and FS 2 are not connected. FS 2 receives subthreshold coherent inputs from a portion of pyramidal cells and subthreshold non-coherent inputs from the other portion of pyramidal cells. It therefore generates only postsynaptic potentials. **(B)** FS 1 and FS 2 are connected through a gap junction containing Cx36 subunit (GJ) and a GABAergic synapse (GABA syn). FS 2 receives subthreshold coherent inputs from the same portion of pyramidal cells as in **(A)**, but it also receives coherent inputs from FS 1 mediated by the GABAergic synapse and the GJ. Summation of those coherent inputs results in rhythmical discharges of FS 2. The activity of pyramidal cells connected with FS 2 receiving non-coherent afferent inputs is attenuated. Notice the different dynamics of potentials mediated through the GJ and GABAergic synapse. Hyperpolarizing phases of GJ potential are slow. They summate, providing a long-lasting decrease in FS 2 membrane excitability and prevent FS 2 burst firing. Contrarily, inhibition provided by the GABAergic synapse between FS cells is very fast, and it precisely harmonizes the activity of FS cells. Alternatively, GABAergic synapses can transmit depolarizing currents to the FS cell when it is activated only at the moderate level and does not discharge (so long as its membrane potential does not achieve -55 mV). The small-amplitude depolarizing phase of potential mediated by the GJ almost coincides with FS 1 spikes.

Fast inhibition provided by GABAergic synapses between interneurons is highly effective in synchronizing PV^+^FS cell networks, in this case meaning the attenuation of non-coherent signals to FS cells (Wang and Buzsáki, 1996; Szabadics et al., 2001; Bartos et al., 2002, 2007). This fast inhibition creates a time-frame for FS cell population activity during oscillations in high frequency bands. Within this time-frame, the time-window when FS-cells are not inhibited followed by the time-window when FS cells are inhibited occur repeatedly. Fast dynamics of these alternations (especially fast during high frequency oscillations) and a very short time-window when FS-cells are not inhibited are the result of very fast inhibitory postsynaptic potentials (IPSPs) kinetics produced specifically by GABAergic synapses connecting FS cells. They are faster than those generated by GABAergic synapses between FS cells and projecting cells (Ali et al., 1999; Bartos et al., 2002; Galarreta and Hestrin, 2002; Pawelzik et al., 2003). It has been suggested that GABAergic fast inhibition is entirely sufficient for fast-frequency activity neuronal synchronization (Hu et al., 2011). Dynamics of inhibition provided by Cx36-GJs is slow. During a train of discharges in FS cells, the long-lasting hyperpolarizing phases of potentials transmitted by GJs undergo temporal summation, decreasing excitation in the FS cell network (Galarreta and Hestrin, 2002; see **Figure 3**).

As was mentioned before, subthreshold excitatory inputs to FS cells result in the excitatory potential mediated by GJs. Interestingly, it was shown in the hippocampal formation that under conditions of moderate level of excitation GABAergic synapses in PV^+^FS cells can produce depolarizing currents (Lamsa and Taira, 2003; Vida et al., 2006; see **Figure 3**). This effect results from the high value of the reversal potential for chloride ions in PV^+^FS interneurons. The value is -55 mV, and in the case of PV^+^FS interneurons it is between the resting and the threshold potential. Therefore, when one FS cell transmits a subthreshold potential through GABAergic synapse to another not very excited FS cell, outward chloride depolarizing currents arise in the GABAergic receptor channels (until the membrane potential will not exceed -55 mV; Lamsa and Taira, 2003; Vida et al., 2006). These data indicate that the nature of the potential transmitted through GABAergic synapses depends on the excitation level in the neuronal network. It seems that GABAergic depolarizing currents can contribute to the field potential gamma and theta rhythm, as during this oscillatory pattern neuronal network excitation is maintained at a moderate level (see **Figure 3**) and some neurons receive subthreshold coherent afferent inputs. Therefore, these subthreshold inputs can be enhanced by GABAergic depolarizing currents transmitted from other FS cells. However, occurrence of GABAergic depolarizing currents inversely coincides with the appearance of high frequency oscillations, which are related to high probability of discharges in neurons.

## POSSIBLE ROLE OF GAP JUNCTIONS IN GENERATION OF HIGH FREQUENCY OSCILLATIONS IN THE HIPPOCAMPAL FORMATION

According to the pioneering hypotheses on GJ role in the brain, it was proposed that GJ transmission underlies high frequency oscillations. In models of the high frequency oscillation mechanism, an exchange of potentials between excitatory neurons through axo-axonal GJs was a putative origin of this oscillatory pattern. In order to test this hypothesis, Traub et al. (2003) prepared minislices containing the stratum oriens isolated from the CA1 area, so that pyramidal cell axons were cut off from their cell bodies and therefore from chemical synaptic inputs to the pyramidal cells. Indeed, kainate application appeared to induce high-frequency oscillations in minislices which proved that these oscillations arise within the axons of pyramidal cells. Gamma-frequency oscillations were not observed in minislices after kainate administration. Interestingly, GABA added to a bath solution in the presence of kainate greatly increased the amplitude and power of high frequency oscillations, while the GABA_A_ receptor antagonist bicuculline abolished them. Field oscillations were also blocked by tetrodoxin and reduced by carbenoxolone. Therefore, the results of this experiment showed that GABAergic transmission is required to evoke high frequency oscillations in the plexus of pyramidal cell axons *in vitro*. It is difficult to interpret the reduction of high frequency oscillations observed in this experiment after carbenoxolone administration, especially considering that the existence of putative GJs between pyramidal cells in the hippocampal CA1 area has not been confirmed (Mercer et al., 2006). Thus, the effect of carbenoxolone could be exerted through non-specific (non-GJ mediated) action.

Most *in vitro* experiments showed that high frequency oscillations in the CA1 and CA3 areas of the hippocampus are sensitive to carbenoxolone (Draguhn et al., 1998; Pais et al., 2003; Traub et al., 2003), octanol (Draguhn et al., 1998; Hormuzdi et al., 2001), or halothane (Draguhn et al., 1998). However, D’Antuono et al. (2005) observed no effect of carbenoxolone or octanol on high frequency oscillations in the dentate gyrus. It is worth mentioning here that local intracortical application of carbenoxolone in anesthetized rats only partially affected high-frequency oscillations (>200 Hz) in the somatosensory cortex (Kamiñski et al., 2011). Discrepancy in the carbenoxolone effect between these data can result from differences in the membrane channels that contribute to the mechanism of high frequency oscillations in particular brain structures (see **Table 2**).

**Table 2:** Effect of non-selective and selective (mediated by Cx36 channels) blockade of gap junction transmission.

Oscillation	Method of GJ blockade	Effect on oscillations	Source
**High frequency oscillations**
*In vitro*	Octanol	Abolishment	Hormuzdi et al. (2001)
	Carbenoxolone, octanol, halothane	Abolishment	Draguhn et al. (1998)
	Carbenoxolone	Abolishment	Pais et al. (2003)
	Carbenoxolone	Reduction	Traub et al. (2003)
	Carbenoxolone, octanol	No effect	D’Antuono et al. (2005)
	Cx36KO	Oscillations occurred less frequently and were slightly slower	Maier et al. (2002)
		No effect	Hormuzdi et al. (2001)
		Oscillations occured only in slices from Cx36KO mice	Pais et al. (2003)
*In vivo*: during wake and SWS sleep	Cx36KO	No effect	Buhl et al. (2003)
**Gamma oscillations**
*In vitro*: transient, persistent	Carbenoxolone	Reduction	Traub et al. (2001)
	Octanol	Abolishment	Traub et al. (2000)
	Cx36KO	Decreased power and frequency	Hormuzdi et al. (2001), Pais et al. (2003)
*In vivo*: during wake	Cx36KO	Decreased, and modulation of gamma power according to the theta phase was disruptted	Buhl et al. (2003)
**Theta oscillations**
*In vitro*	Carbenoxolone	Abolishment	Konopacki et al. (2004)
*In vivo*: during wake	Cx36KO	Larger portion of theta rhythm shifted to lower theta frequencies	Allen et al. (2011)

Contrary to the above presented experiments, the results of several studies where GJ coupling between neurons was specifically blocked altogether indicate that Cx36-GJs are not required for the neuronal synchronization underlying high frequency oscillations. While Maier et al. (2002) observed that ripple frequency oscillations occurred less frequently and were slightly slower in the CA1 area in brain slices from Cx36KO mice, Pais et al. (2003) noticed ripple-like activity only in the brain slices from Cx36KO mice, but not in wild-type slices. Hormuzdi et al. (2001) reported no differences in high frequency oscillations in the CA3 area in slices from wild-type and Cx36KO mice. Results obtained *in vitro* are in accordance with *in vivo* data. High frequency oscillations recorded in freely moving Cx36KO mice from the hippocampal CA1 area during silent wake as well as those recorded during slow wave sleep were not altered in comparison to wild-type animals (Buhl et al., 2003; see **Table 2**).

High frequency oscillations (ripples) coincide with a large increase in population activity, including pyramidal cells and interneurons (Csicsvari et al., 1998, 1999; Traub and Bibbig, 2000; Biró and Nusser, 2005). During high frequency oscillations field potential oscillatory pattern PV^+^FS cells can discharge in each cycle of oscillation, i.e., they discharge with high frequency up to 200 Hz. As was described before, when FS cells discharge, a main part of the potential transmitted through GJs to the coupled cells is hyperpolarization. Moreover, temporal summation of long-lasting hyperpolarization transmitted through GJs could decrease the excitation level within the FS cell network, thus decreasing the frequency of PV^+^FS cell firing. I suggest that the inhibitory influence of GJ transmission could impair fast firing of PV^+^FS cells during high frequency oscillations. Interestingly, it was demonstrated that changes in cell excitation levels have a modulatory effect on the synaptic strength of electrical synapses (Usher et al., 1999; Alvarez et al., 2002; Kothmann et al., 2007, 2012; Zsiros and Maccaferri, 2008; Vervaeke et al., 2010; Haas et al., 2011; Otsuka and Kawaguchi, 2013; Pereda et al., 2013). Several studies suggest that activity-dependent plasticity of GJs may play a role in shaping behaviorally relevant functional states within the brain (Usher et al., 1999; Garcia-Rill et al., 2007; Kothmann et al., 2007, 2012; Vervaeke et al., 2010; Haas et al., 2011; Haas and Landisman, 2012). In the hippocampal formation, increased interneuron excitation levels can reduce the synaptic strength of electrical synapses (Zsiros and Maccaferri, 2008). I would expect that a lack of the effect of neuronal GJ blockade on high frequency oscillations *in vitro* (Hormuzdi et al., 2001; Pais et al., 2003; D’Antuono et al., 2005) and *in vivo* (Buhl et al., 2003) results from a natural decrease of the GJ transmission level occurring alongside an increase of neuronal activity during this rhythmical pattern (Zsiros and Maccaferri, 2008). These data suggest that fast inhibition provided by FS interneurons, but not Cx36-GJ transmission, is of crucial significance for the mechanism of high frequency oscillations.

## POSSIBLE ROLE OF GAP JUNCTIONS IN GENERATION OF GAMMA AND THETA OSCILLATIONS IN THE HIPPOCAMPAL FORMATION

In *in vitro* studies, carbenoxolone disrupted transient gamma-frequency oscillations (Traub et al., 2001), while persistent gamma-frequency oscillations were suppressed by octanol (Traub et al., 2000). Carbenoxolone also abolished theta oscillations in the hippocampal CA1 area in rat brain slices (Konopacki et al., 2004; see **Table 2**). Similar results were obtained *in vivo* in anesthetized rats after local application of carbenoxolone (Bocian et al., 2009), and during prolonged recordings it was shown there that the effect of carbenoxolone is reversible after a few hours. Bissiere et al. (2011) assessed the effect of carbenoxolone-induced GJ inhibition on behavioral test performance as well as on field theta oscillations in mice. Carbenoxolone attenuated field theta rhythm power and disrupted context-dependent fear learning. However, the results of the experiments with non-specific blockade by carbenoxolone do not provide exact information whether the observed inhibitory effect on gamma and theta oscillations was due to blockade of neuronal GJs, glial GJs, or both or even by action on other membrane channels.

Experiments with selective blockade of Cx36-GJ transmission provide a more detailed insight into the contribution of neuronal GJ to neuronal synchronization underlying gamma and theta rhythms. Persistent gamma rhythm appeared with decreased power and frequency in brain slices from Cx36KO mice in comparison to slices from wild-type animals (Hormuzdi et al., 2001; Pais et al., 2003). Persistent gamma rhythm *in vitro* is induced by muscarinic or kainate receptor activation, thus it resembles wake activity-related gamma rhythm. Therefore, the results from *in vitro* experiments seem to be consistent with *in vivo* data. In freely moving Cx36KO mice the power of gamma rhythm recorded during motor activity decreased, and modulation of gamma power according to the theta phase was affected in comparison to wild-type animals. However, gamma rhythm occurring during REM sleep was not altered in Cx36KO mice (Buhl et al., 2003).

In their experiment, Buhl et al. (2003) also analyzed theta rhythm in freely moving Cx36KO mice. Both types of theta rhythm, occurring during motor activity and REM phase sleep, were not altered in Cx36KO mice in comparison to wild-types. Buhl et al. (2003) recorded the phase relationship of unit discharges to theta rhythm. While some tendency of pyramidal cells to discharge in a slightly later phase of the theta cycle in Cx36KO mice was seen, differences between groups were not significant. Allen et al. (2011) performed field and unit recordings in Cx36KO mice that underwent learning training based on tasks related to neuronal coding of spatial information. While both Cx36KO and wild-type mice presented prominent theta oscillations during spatial exploration, a larger portion of theta rhythm in Cx36KO mice was shifted to lower theta frequencies than in wild-type controls. Moreover, pyramidal cells identified as place cells in Cx36KO mice have shown lower spatial selectivity than in a control group. They responded to a higher number of fields within the explored spatial area and their receptive fields were larger than in wild-type mice. Place cells discharged at later phases of theta cycle in Cx36KO mice than in control mice. The discrepancy between these two *in vivo* studies (Buhl et al., 2003; Allen et al., 2011) can be explained on the grounds of the data analysis method. While Buhl et al. (2003) assessed changes in the oscillations by theta power, Allen et al. (2011) analyzed peak frequency in the theta band and the power of this peak frequency in the power histogram. Therefore, Allen observed that while the theta power was not changed in Cx36KO vs. wild-type animals, the prominent oscillation frequency (within the theta frequencies band) was different. In the case of unit discharges, Buhl et al. (2003) combined neurons recorded during wheel running with those recorded during REM phase of sleep, and differences between Cx36KO and wild-type groups were analyzed on the basis of these combined data. Therefore, possible differences in theta oscillations and theta phase-relationship of unit discharges during active behavior elucidated by Allen et al. (2011) could have been missed in the work of Buhl et al. (2003). The final conclusion is that Cx36-GJs contribute to theta rhythm occurring during active behavior (see **Table 2**).

In sum, Cx36-GJs contribute most strongly to active behavior-related gamma oscillations. While they are also involved in the active behavior-related theta rhythm to a lesser degree, they are still required for proper timing of neuronal discharges and reliability of information coding in the neuronal network. The mechanism of GJ influence on time order of neuronal discharges is probably based on their participation in the mechanism of synchrony detection as they enhance simultaneous inputs, and in cooperation with GABAergic synapses, inhibit delayed ones (**Figure 3**). Another important contribution of GJs to neuronal discharge timing could be the direct exchange of excitatory potentials between FS interneurons in response to subthreshold excitatory inputs. As a result, more interneurons may generate rhythmical activity during oscillations, imposing time order on more pyramidal cells (**Figure 3**). The specific action of GJs during gamma oscillation concerns their modulation of neuronal network excitability, as during train of discharges in FS cells, the long-lasting hyperpolarizing phases of potentials transmitted by GJs undergo temporal summation, decreasing excitation in the network of FS cells and preventing burst firing in interneurons (Galarreta and Hestrin, 2002; **Figure 3**).

While the described way of GJ action may contribute to the precision of neuronal discharges and seems to be especially important during states of active behavior when a lot of noise invade neuronal networks, it raises the question of why GJ’s should not be involved in high frequency oscillations, as they are related to high activation in neuronal networks. In the model I suggest that inhibition resulting from long-lasting hyperpolarizing phases of potentials transmitted by GJs, when FS cells generate a train of discharges, could prevent high frequency discharges in FS cells, and thus high frequency oscillations in the neuronal network. Also, it is quite probable that the order of neuronal discharges during high frequency oscillations, which occur during silent wake or the SWS phase of sleep (i.e., after active behavior), could be determined by synaptic changes that the neuronal network has undergone before, during active behavior (i.e., changes related to memory trace formation). A similar explanation could be relevant for small GJ contribution to REM sleep-related gamma and theta rhythm. However, more experiments are required to answer whether GJs take part in the mechanism of these oscillations.

## POSSIBLE ROLE OF GAP JUNCTIONS IN GENERATION OF EPILEPTIFORM ACTIVITY IN THE HIPPOCAMPAL FORMATION

An intriguing issue is the role of GJs in epileptiform discharges when neuronal networks undergo hyperexcitability. For years GJ transmission was regarded as a source of large neuronal synchronization during seizure. It seemed that blockage of the GJ coupling between neurons would have a protective effect against epileptiform activity. The great majority of research on the role of GJs in epilepsy, to date, has been performed using non-specific GJ uncouplers, such as carbenoxolone and octanol (Perez-Velazquez et al., 1994; Carlen et al., 2000; Ross et al., 2000; Köhling et al., 2001; Jahromi et al., 2002; Gajda et al., 2003, 2006; Nilsen et al., 2006; Bostanci and Bağirici, 2007). In these studies, application of non-specific GJ blockers decreased the frequency and/or amplitude of epileptiform spikes in the field potential (Ross et al., 2000; Köhling et al., 2001; Jahromi et al., 2002; Bostanci and Bağirici, 2007) or decreased the duration of seizure epochs (Gajda et al., 2003, 2006; Nilsen et al., 2006). In one *in vitro* study seizure activity was suppressed in the field potential after application of octanol (Perez-Velazquez et al., 1994). While the results of these experiments do not provide an answer to whether glial and/or neuronal GJs play a role in epileptiform activity, data from a few studies using selective blockade of neuronal GJs by quinine or mefloquine showed that neuronal GJs do not contribute to hyperexcitation underlying epileptiform discharges in the hippocampus and neocortex (Gajda et al., 2005; Behrens et al., 2011) or in the neocortical slices (Voss et al., 2009, 2010). Behrens et al. (2011) showed that mefloquine-induced Cx36 channel blockade did not affect epileptiform discharges in the hippocampal area CA3 *in vitro*. In the *in vivo* study by Gajda et al. (2005), quinine application decreased the duration of seizures evoked by 4-aminopyridine (4-AP), but significantly increased their number in the rat neocortex. Moreover, a new seizure component characterized by the lowest amplitude but highest frequency appeared during quinine treatment despite 4-AP induced discharge patterns. Another effect of quinine was the significant amplitude increase of discharges in the 11–12 Hz frequency band. While quinine and mefloquine have also been shown to act on potassium channels, decrease the frequency of pyramidal cell firing, and elongate afterhyperpolarization following action potential (Smirnov et al., 1999; Päsler et al., 2007; Behrens et al., 2011), it seems that these effects would rather have an attenuating effect on the epileptiform activity. Therefore, on the basis of these experiments it could be assumed that Cx36-GJ blockade does not prevent neuronal networks from synchronization related to epileptiform discharges.

At least two things need be taken into consideration when trying to understand the minor effect of Cx36-GJs blockade on epileptiform activity. First, an increase of intracellular pH occurs at the onset of epileptiform bursts (Chesler, 2003; Sinning and Hübner, 2013). As molecular investigations by González-Nieto et al. (2008) indicate, channels containing Cx36 subunits show opposite response to changes in the levels of intracellular pH then other connexin channels. Alkalization reduces conductance through Cx36 channels (González-Nieto et al., 2008). Second, high neuronal activity is related to a decrease of Cx36-GJ conductance (Usher et al., 1999; Alvarez et al., 2002; Kothmann et al., 2007, 2012; Zsiros and Maccaferri, 2008; Vervaeke et al., 2010; Haas et al., 2011; Otsuka and Kawaguchi, 2013). Therefore, it seems that neuronal GJs do not play an important role in development and maintenance of epileptiform activity.

## CONCLUSION

Transmission mediated by GJs containing Cx36 subunits appears particularly important for gamma and theta rhythm generated in the hippocampal formation during wakefulness. Cx36-GJs, in cooperation with GABAergic synapses within FS interneuron network, contribute to the time-precision of neuronal discharges through the mechanism of synchrony detection (Galarreta and Hestrin, 2001). Cx36-GJs, in parallel with GABAergic synapses, enhance simultaneous and attenuate delayed inputs during oscillatory population activity in gamma and theta frequency bands. In the model proposed here, the specific Cx36-GJ contribution to gamma rhythm is in preventing FS cells from burst activity during trains of discharges. Due to the influence of Cx36-GJs on neuronal discharge timing, their contribution to information processing during wake activity-related field potential gamma and theta rhythm could be significant. Indeed, it was demonstrated that Cx36 subunit knockout (Allen et al., 2011; Postma et al., 2011; Wang and Belousov, 2011) resulted in learning impairment and affected neuronal plasticity.

In contrast, Cx36-GJs seem to play minor role in the mechanism of high frequency oscillations generated during states of silent wake or slow wave sleep. Data indicate that alongside the increase of neuronal activity which accompanies field potential high frequency oscillations, the level of transmission through Cx36-GJ channels decreases. I propose that another function of GJs in neurons is to regulate the level of direct intercellular communication in response to intracellular signals (including those related to the level of cellular activity), which is a general role of GJs in the majority of bodily tissues. However, the adjustments of Cx36-GJs to the specific functional requirements of neuronal networks in the brain are GJ presence specific for a particular group of interneurons in networks, and low level of the conductance in GJ channels formed by the Cx36 subunit.

## Conflict of Interest Statement

The author declares that the research was conducted in the absence of any commercial or financial relationships that could be construed as a potential conflict of interest.
